# Additional regulatory activities of MrkH for the transcriptional expression of the *Klebsiella pneumoniae mrk* genes: Antagonist of H-NS and repressor

**DOI:** 10.1371/journal.pone.0173285

**Published:** 2017-03-09

**Authors:** Miguel A. Ares, José L. Fernández-Vázquez, Sabino Pacheco, Verónica I. Martínez-Santos, Ma. Dolores Jarillo-Quijada, Javier Torres, María D. Alcántar-Curiel, Jorge A. González-y-Merchand, Miguel A. De la Cruz

**Affiliations:** 1 Unidad de Investigación Médica en Enfermedades Infecciosas y Parasitarias, Hospital de Pediatría, Centro Médico Nacional Siglo XXI-IMSS, Ciudad de México, México; 2 Departamento de Microbiología, Escuela Nacional de Ciencias Biológicas, Instituto Politécnico Nacional, Ciudad de México, México; 3 Unidad de Investigación en Medicina Experimental, Facultad de Medicina, Universidad Nacional Autónoma de México, Ciudad de México, México; 4 Departamento de Microbiología Molecular, Instituto de Biotecnología, Universidad Nacional Autónoma de México, Cuernavaca, México; 5 Unidad Académica de Ciencias Químico Biológicas, Universidad Autónoma de Guerrero, Chilpancingo, Guerrero, México; Centre National de la Recherche Scientifique, Aix-Marseille Université, FRANCE

## Abstract

*Klebsiella pneumoniae* is a common opportunistic pathogen causing nosocomial infections. One of the main virulence determinants of *K*. *pneumoniae* is the type 3 pilus (T3P). T3P helps the bacterial interaction to both abiotic and biotic surfaces and it is crucial for the biofilm formation. T3P is genetically organized in three transcriptional units: the *mrkABCDF* polycistronic operon, the *mrkHI* bicistronic operon and the *mrkJ* gene. MrkH is a regulatory protein encoded in the *mrkHI* operon, which positively regulates the *mrkA* pilin gene and its own expression. In contrast, the H-NS nucleoid protein represses the transcriptional expression of T3P. Here we reported that MrkH and H-NS positively and negatively regulate *mrkJ* expression, respectively, by binding to the promoter of *mrkJ*. MrkH protein recognized a sequence located at position -63.5 relative to the transcriptional start site of *mrkJ* gene. Interestingly, our results show that, in addition to its known function as classic transcriptional activator, MrkH also positively controls the expression of *mrk* genes by acting as an anti-repressor of H-NS; moreover, our results support the notion that high levels of MrkH repress T3P expression. Our data provide new insights about the complex regulatory role of the MrkH protein on the transcriptional control of T3P in *K*. *pneumoniae*.

## Introduction

*Klebsiella pneumoniae* is an opportunistic Gram-negative bacterium causing nosocomial infections ranging from pneumonia and urinary tract infections to septicemia and pyogenic liver abscesses [1–6]. Several virulence determinants of *K*. *pneumoniae* have been described: capsular polysaccharide, lipopolysaccharide, siderophores and pili [1, 7, 8]. Different types of pili are encoded in the genome of *K*. *pneumoniae* such as Type 1 pilus (T1P), Type 3 pilus (T3P) and *E*. *coli* common pilus (ECP) [9–12]. In particular, *K*. *pneumoniae* T3P mediates adherence to renal tubular cells and cells of the respiratory tract such as tracheal epithelial cells, and basolateral surfaces of lung tissue, which is crucial for biofilm formation [13–17].

T3P is genetically organized in three transcriptional units: the *mrkABCDF* polycistronic operon, the *mrkHI* bicistronic operon and the *mrkJ* gene. The biogenesis of T3P is dependent on the *mrkABCDF* operon expression [18, 19]. The filament is composed of the major pilus subunit MrkA and the tip adhesion protein MrkD [8]. MrkH is a regulatory protein encoded in the *mrkHI* operon, which positively regulates the *mrkA* pilin gene and its own expression [20–22]. MrkH protein contains a PilZ domain, whose interaction with c-di-GMP is crucial for its role as a transcriptional activator [23]. The *mrkHI* operon also codes for MrkI, a LuxR-type transcriptional regulator reported to act as a co-activator for the expression of *mrkA* [20, 24]. The *mrkJ* gene encodes a phosphodiesterase that degrades c-di-GMP, which in turn, controls the MrkH activity [25].

In addition to MrkH, global regulators such as the H-NS nucleoid protein also control the T3P expression [26]. H-NS is a DNA-binding protein, which plays a dual role as an architectural protein component of the nucleoid and as a global regulator of bacterial gene expression [27, 28]. H-NS affects bacterial evolution by directly repressing the expression of AT-rich DNA (i.e. pathogenicity islands) acquired by horizontal transfer events, thus facilitating tolerance of these foreign sequences, which allows their integration into pre-existing regulatory networks [29–31]. H-NS differentially regulates the transcriptional expression of T3P: represses *mrkHI/mrkJ* and activates *mrkA* [26].

In this work we reported that the *mrkJ* gene is directly activated and repressed by MrkH and H-NS, respectively. A sequence located at position -63.5 relative to the transcriptional start site of *mrkJ* gene was recognized by the MrkH protein. Furthermore, we found that MrkH induces the expression of *mrkJ*, as well as that of *mrkI*, by dual regulation: it antagonizes H-NS-mediated repression on these genes and also acts as a transcriptional activator. Moreover, our results support the notion that MrkH can also act as a transcriptional repressor of *mrk* genes. Overall, our data provides new insights on the complex regulatory function of MrkH protein on the transcriptional control of T3P in *K*. *pneumoniae*.

## Materials and methods

### Bacterial strains and culture conditions

Bacterial strains and plasmids used in this study are listed in Table 1. Bacterial cultures were grown in Luria-Bertani (LB) broth until exponential phase (OD_600nm_ = 0.8) was reached. Cultures were grown overnight at 37°C shaken at 160 rpm, with or without antibiotics [200 μg/ml (ampicillin), 50 μg/ml (kanamycin), 30 μg/ml (chloramphenicol) or 10 μg/ml (tetracycline)]. MrkH production from pT6-MrkH plasmid was induced with different L(+)-arabinose concentrations under several genetic backgrounds.

**Table 1:** Bacterial strains and plasmids used in this study.

Strain or plasmid	Genotype or description	Reference or source
***K*. *pneumoniae* strains**		
*Kpn* 123/01	WT, serotype K39	[26]
*Kpn hns*	Δ*hns*::Km^R^	[26]
*Kpn mrkH*	Δ*mrkH*::Km^R^	This study
*Kpn mrkH*	Δ*mrkH*::FRT	This study
*Kpn hns mrkH*	Δ*hns*::Km^R^ Δ*mrkH*::Cm^R^	This study
*Kpn mrkJ*	Δ*mrkJ*::Km^R^	This study
*Kpn hns mrkJ*	Δ*hns*::Km^R^ Δ*mrkJ*::Cm^R^	This study
*Kpn mrkI*	Δ*mrkI*::Km^R^	This study
Kpn mrkJ*	*mrkJ*- ΔMrkHbox::FRT	This study
		
**Plasmids**		
pMPM-T6	p15A derivative cloning vector, pBAD (*ara*) promoter, Tc^R^	[45]
pT6-MrkH	pMPM-T6 derivative expressing MrkH-His_6_ from the pBAD (*ara*) promoter	[26]
pKK-*mrkJ*-wt	pKK232-9 derivative containing a *mrkJ-cat* transcriptional fusion from nucleotides -352 to +33	This study
pKK-*mrkJ*-mut	pKK-*mrkJ*-wt derivative containing three point mutations in the TAT motif of the MrkH-binding box	This study
pKD119	pINT-ts derivative containing the λ Red recombinase system under an arabinose-inducible promoter, Tc^R^	[32]
pKD4	pANTsγ derivative template plasmid containing the kanamycin cassette for λ Red recombination, Ap^R^	[32]
pKD3	pANTsγ derivative template plasmid containing the chloramphenicol cassette for λ Red recombination, Ap^R^	[32]

Ap^R^, ampicillin resistance; Km^R^, kanamycin resistance; Cm^R^, chloramphenicol resistance; Tc^R^, tetracycline resistance.

### Construction of *K*. *pneumoniae* mutants and transcriptional fusions

Construction of single and double mutants was performed as previously described [26]. We generated a Δ*mrkH* mutant, by amplifying a PCR product containing *mrkH* sequence flanking a kanamycin cassette using the pKD4 plasmid, and using gene-specific primer pairs (Table 2). Kpn mrkJ* mutant was obtained by deletion of the MrkH-box on the *mrkJ* promoter (*mrkJ*-ΔMrkHbox::Km^R^) using Kpn-mrkJΔMrkH-H1P1 and Kpn-mrkJΔMrkH-H2P2 primers (Table 2). The FRT-flanked Km cassette was excised from strains Δ*mrkH* and ΔmrkJ* after transformation with pCP20, as described previously [32]. For Δ*hns* Δ*mrkH* and Δ*hns* Δ*mrkJ* double mutants, *K*. *pneumoniae* Δ*hns* was targeted to carry out the mutagenesis of *mrkH* and *mrkJ*, amplifying a PCR fragment containing *mrkH* and *mrkJ* sequences flanking a chloramphenicol cassette using the pKD3 plasmid. The corresponding mutations were confirmed by PCR and sequencing.

**Table 2:** Primers used in this study.

Primer	Sequence (5’-3’)	Target gene
**For qPCR**		
cat-F	TGGCAATGAAAGACGGTGAG	*cat*
cat-R	AGAAACTGCCGGAAATCGTC	
		
**For mutagenesis**		
Kpn-mrkH-H1P1	CACGACAACTATTTACAAGGGATGCA TATGACAGAGGGAACGATATGTAGGC TGGAGCTGCTTCG	*mrkH*
Kpn-mrkH-H2P2	GCAATATACTGTCCAAGGTTGTCAGA TTCTCTTTTTGCGCTTGGCCATATGA ATATCCTCCTTAG	
Kpn-mrkI-H1P1	CAAAAAGAGAATCTGACAACCTTGGA CAGTATATTGCTGTACACCTGTAGGC TGGAGCTGCTTCG	*mrkI*
Kpn-mrkI-H2P2	ACTGATTTACCGGGAGAACATTTAGC ATTGATGGAGAGCGGCAATCATATGA ATATCCTCCTTAG	
Kpn-mrkJ-H1P1	CTAACCTCGTGAAGAGGGATAATGAA CACTAAAATATTCGAAGACTGTAGGC TGGAGCTGCTTCG	*mrkJ*
Kpn-mrkJ-H2P2	GCCGGGAATTCCCGGCTTTGTTTACA TGGCAATATCATCGGCGACCATATGA ATATCCTCCTTAG	
Kpn-mrkJΔMrkH-H1P1	ATGCTAAATGTTCTCCCGGTAAATCA GTAGCGGATAAAGCGTACTTGTAGGC TGGAGCTGCTTCG	*mrkJ*
Kpn-mrkJΔMrkH-H2P2	ACCTGATGATTAATGGGAATGGCGGG AAATGTAAATCAACAGCGACATATGA ATATCCTCCTTAG	
**For mutants characterization**		
Kpn-mrkH-F	CTATTGCTATAAGAAAAATCAAAC	*mrkH*
Kpn-mrkH-R	TGATAGATTGAGTGACCAATGAGA	
Kpn-mrkI-F	TAGAGAAGATACTGCTGGACCTGA	*mrkI*
Kpn-mrkI-R	GGAATGGCGGGAAATGTAAATCA	
Kpn-mrkJ-F	CGCCATTCCCATTAATCATCAGG	*mrkJ*
Kpn-mrkJ-R	TACCAGCTGGGCAACGTG	
		
**For constructions**		
mrkJ-BamHI-F	ACT*GGATCC*TCATCTATCGTCCAGCGCGCC	*mrkJ*
mrkJ-BamHI-R	CAT*AAGCTT*TCTTCACGAGGTTAGTCAGAC	
mrkJ-mut-F	TACTCGCTCGCTGTTGATTTACATTTCCCGC	
mrkJ-mut-R	CGGGAAATGTAAATCAACAGCGAGCGAGTA	
		
**For EMSA**		
mrkJ-EM-F	ACTGGCCCAGACGATTATTTTC	*mrkJ*
mrkJ-EM-R	TAAAATGTTGTCTTCGAATATTTTAG	
mrkH-EM-F	AGGCGCAGGAGTTGAACGAGGTC	*mrkH*
mrkH-EM-R	GGTCTTTATCGTTCCCTCTGTCATATG	
mrkA-EM-F	ATGGCGGTTTGATGGCGTAAAC	*mrkA*
mrkA-EM-R	TGCTGCAGAGAGAAGAACCTTTTTC	
fbpA-EM-F	TTCCTGACCAGCGAGCTGCCG	*fbpA*
fbpA-EM-R	CCCCAGTACTCCCAGCTGTGC	

Italic letters indicate the respective restriction enzyme site in the primer. The sequence corresponding to the template plasmids pKD4 or pKD3 is underlined.

Regulatory region of *mrkJ* was amplified using primers mrkJ-BamHI-F and mrkJ-HindIII-R (Table 2). This product was digested with BamHI and HindIII and then ligated into pKK-232-8 (Ap^R^), previously digested with the same restriction enzymes. This plasmid was digested with BamHI and NcoI and the insert was subcloned into pKK-232-9 plasmid (Km^R^) [33] generating pKK-*mrkJ*-wt construct. Site-directed mutagenesis was carried out on the pKK-*mrkJ*-wt plasmid by overlapping PCR with specific primers (mrkJ-mut-F and mrkJ-mut-R) to obtain the pKK-*mrkJ*-mut using the primers mrkJ-mut-F and mrkJ-mut-R (Table 2). Plasmids were sequenced to verify the integrity of the inserts and the introduction of the point mutations.

### Quantitative RT-PCR

Total RNA extraction was performed using the hot phenol method [34]. Purification of RNA and qRT-PCR were performed as previously reported [26]. 16S rRNA was used as a reference gene for normalization and the relative gene expression was calculated using the 2^-ΔCt^ method [35]. Primers for qPCR experiments were previously reported [26], except for *cat* quantification (Table 2).

### MrkH-His_6_ purification

Purification of MrkH-His_6_ protein was performed with Ni-nitrilotriacetic acid. Briefly, *K*. *pneumoniae* carrying the pT6-MrkH (Table 1) was grown to mid-logarithmic phase. L(+)-arabinose (Sigma-Aldrich) was added to a final concentration of 0.1%, and bacteria were grown for 6 h at 30°C. Cells were then pelleted by centrifugation, resuspended in urea buffer [8M urea, 100mM NaH_2_PO_4_, 10mM Tris-HCl (pH 8.0)] and disrupted by sonication. The suspension was centrifuged, and the supernatant was filtered through a Ni-nitrilotriacetic acid agarose column (QIAExpress, Qiagen) preequilibrated with urea buffer. After an extensive washing with binding buffer containing 50mM imidazole (100 ml), protein was eluted with 500mM imidazole. Fractions were analyzed by SDS-PAGE, and protein concentration was determined by the Bradford procedure. Aliquots of the purified protein were stored at -70°C until used.

### Electrophoretic Mobility Shift Assay (EMSA)

EMSA experiments were performed as previously described [36, 37]. PCR products corresponding to the *mrk* promoter regions were amplified using specific primers (Table 2). PCR products (100 ng) were mixed with increasing concentrations of H-NS-*Myc*-His_6_ or MrkH-His_6_ in the presence of the binding buffer 10X (400mM HEPES, 80mM MgCl_2_, 500mM KCl, 10mM DTT, 0.5% NP40 and 1 mg/ml BSA). *fbpA* coding region of *Mycobacterium tuberculosis* was used as negative control. The reactions were incubated during 30 min at room temperature (for H-NS) and 4°C (for MrkH), and then separated in 6% SDS-PAGE gels in Tris-Borate-EDTA buffer. The DNA bands were visualized by the ethidium bromide staining.

### Assay for biofilm formation on abiotic surface

Adhesion to abiotic surface (polystyrene) was analyzed using 96-well plates as described previously [26]. Overnight cultures of bacteria grown in LB broth (10 μl) were added to 1 ml of LB. This volume was distributed in quintuples (200 μl per well) into a 96-well plate and incubated at room temperature for 24 h. Unbound bacteria were removed by washing the wells three times with PBS, and bound bacteria were stained with 1% violet crystal (CV) for 20 min. Wells were thoroughly rinsed thrice with PBS, and the dye in the adhered bacteria was solubilized with 100 μl of ethanol 70%. Finally, the amount of extracted violet crystal was determined using an enzyme-linked immunosorbent assay (ELISA) and measuring the OD_600_ in a multiskan plate reader (Thermo Scientific).

### Statistical analysis

For statistical differences, one-way ANOVA followed by the Tukey’s comparison test was performed using Prism 5.0 (GraphPad Software Inc., San Diego, CA, USA). *p*≤0.05 was considered statistically significant.

## Results

### *mrkJ* promoter is directly regulated by MrkH

**Fig 1:**
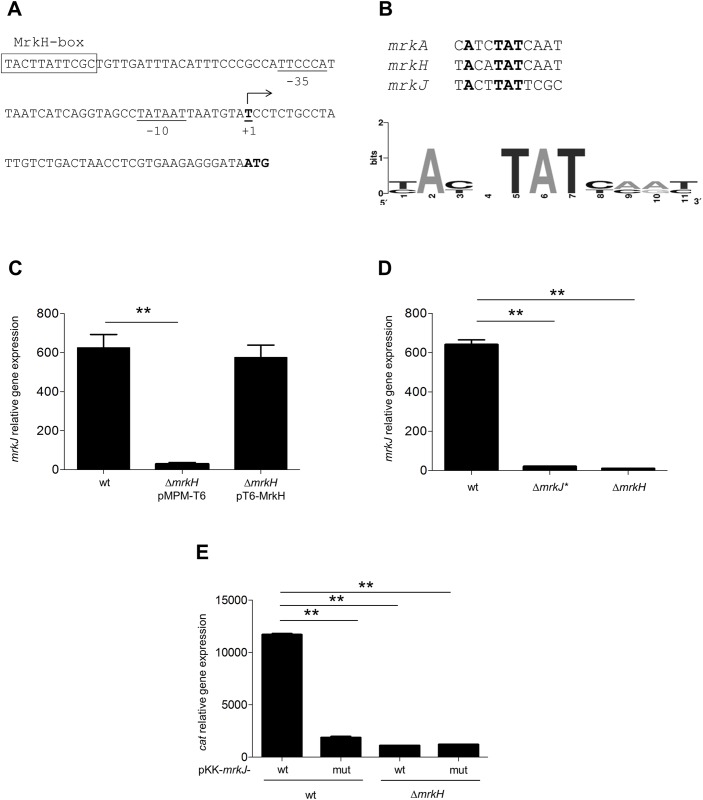
MrkH regulates the *mrkJ* promoter. A. Schematic representation of the *mrkJ* promoter. The panel shows the nucleotide sequence of the regulatory region, showing the previously reported transcription start site (+1) [24]. The -35 and -10 promoter sequences and the transcription start site are underlined. Putative MrkH-binding site is boxed. B. Logo motif analysis using the MrkH-binding sites for *mrkA*, *mrkH* and *mrkJ* promoter regions. C. Transcriptional expression (qRT-PCR) of *mrkJ* gene in WT, Δ*mrkH* and complemented Δ*mrkH* backgrounds. D. *mrkJ* expression (qRT-PCR) in the wild-type (WT) and *mrkJ*ΔMrkHbox::FRT (mrkJ*). E. qRT-PCR assays determining the *cat* expression of *mrkJ* (pKK-*mrkJ*-wt) and a mutant variant within the MrkH-binding box (pKK-*mrkJ*-mut). Results represent mean and standard deviations of three independent experiments. ns, not significant; **, statistically significant with respect to the WT strain (*p*<0.01).

Although MrkH protein has been reported as a master regulator of the T3P [20–22], there are no reports of its effect on *mrkJ* expression; thus we studied whether the MrkH DNA-binding sequence, previously reported for *mrkA* and *mrkH* [21, 22] was present in the regulatory region of *mrkJ*. We found a putative MrkH-box on the *mrkJ* promoter, located at position -63.5 relative to its transcriptional start site (Fig 1A). This putative MrkH binding sequence presented the TAT motif conserved in the MrkH binding sites on *mrkA* and *mrkH* (Fig 1B). Then, to know whether MrkH regulates the expression of *mrkJ*, we determined the expression of this gene in the wild-type (WT) *K*. *pneumoniae* strain and its isogenic Δ*mrkH* mutant. As shown in Fig 1C, the transcription level of *mrkJ* was drastically decreased in the Δ*mrkH* mutant, with respect to the WT strain. The complemented Δ*mrkH* mutant had expression levels similar to the WT strain. To demonstrate that the putative MrkH-box was essential for MrkH-mediated *mrkJ* activation, the TACTTATTCGC sequence (Fig 1A) was deleted from the *K*. *pneumoniae* chromosome to generate a mutant strain, Kpn mrkJ* (*mrkJ*-ΔMrkHbox::FRT). We found that this deletion caused a severe reduction in the transcription of *mrkJ* gene (Fig 1D). Moreover, this reduction was similar to that observed in the absence of MrkH, supporting the notion that the deleted sequence is essential for the MrkH-mediated activation of the *mrkJ* promoter. In addition, using transcriptional reporters, we cloned the regulatory region of *mrkJ* and introduced a three nucleotides change in the TAT conserved motif into MrkH box (TAT to CGC). This mutant construction presented a reduction in MrkH-mediated *mrkJ* activation (Fig 1E), corroborating the relevance of the TAT motif in the MrkH-mediated *mrkJ* positive regulation. These results indicate that, similarly to other *mrk* genes, MrkH positively regulates the expression of *mrkJ*.

### MrkH induces the expression of *mrkJ* by antagonizing H-NS-mediated repression and by acting as a transcriptional activator

**Fig 2:**
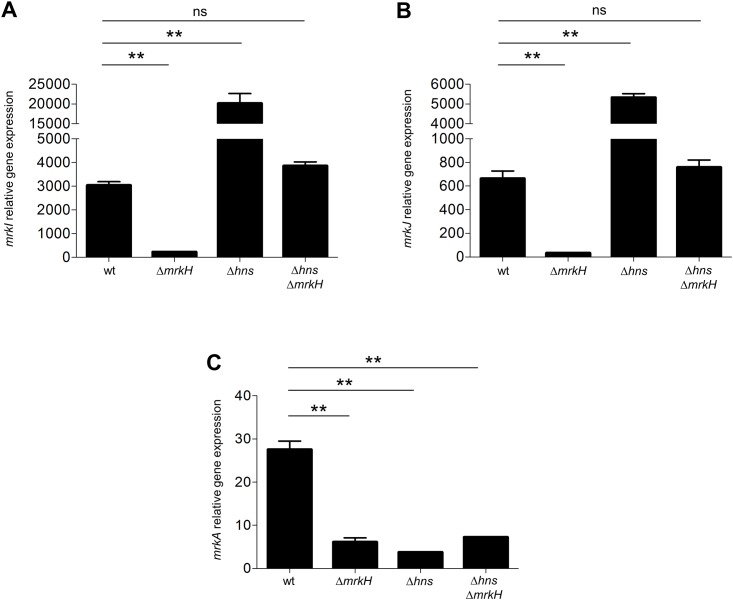
MrkH acts as anti-repressor and activator of *mrk* genes. Transcriptional expression (qRT-PCR) of *mrkI* (A), *mrkJ* (B) and *mrkA* (C) genes in the WT, Δ*mrkH* mutant, Δ*hns* mutant and Δ*hns* Δ*mrkH* double mutant. Results represent the mean and standard deviations of three independent experiments. ns, not significant; **, statistically significant with respect to the WT strain (*p*<0.01).

We have previously reported that H-NS represses the transcription of *mrkH*, *mrkI* and *mrkJ* [26]. Since our results indicated that the expression of the *mrkJ* gene is positively and negatively controlled by MrkH and H-NS, respectively, we hypothesized that MrkH induces the expression of these genes by counteracting the H-NS mediated repression. To investigate this, we determined the expression of the *mrkI* and *mrkJ* genes in a Δ*hns* Δ*mrkH* double mutant, by qRT-PCR (Fig 2A and 2B). The expression of the *mrkA* gene was also tested as a control. As expected, the expression of the *mrkA* gene was reduced in the Δ*hns* Δ*mrkH* double mutant, at levels similar to those observed in the Δ*hns* and Δ*mrkH* single mutants (Fig 2C). Interestingly, the expression of *mrkI* and *mrkJ* was restored in the Δ*hns* Δ*mrkH* double mutant, to a level similar to that in the WT strain, which supports that MrkH induces the expression of both genes by antagonizing their H-NS-mediated repression. However, the expression levels of the *mrkI* and *mrkJ* genes in the Δ*hns* Δ*mrkH* double mutant were lower than those observed in the Δ*hns* mutant (Fig 2A and 2B), suggesting that MrkH further activates the expression of these genes in the absence of H-NS, showing a dual activator/anti-repressor activity.

### MrkH and H-NS proteins directly bind to the *mrk* promoters

**Fig 3:**
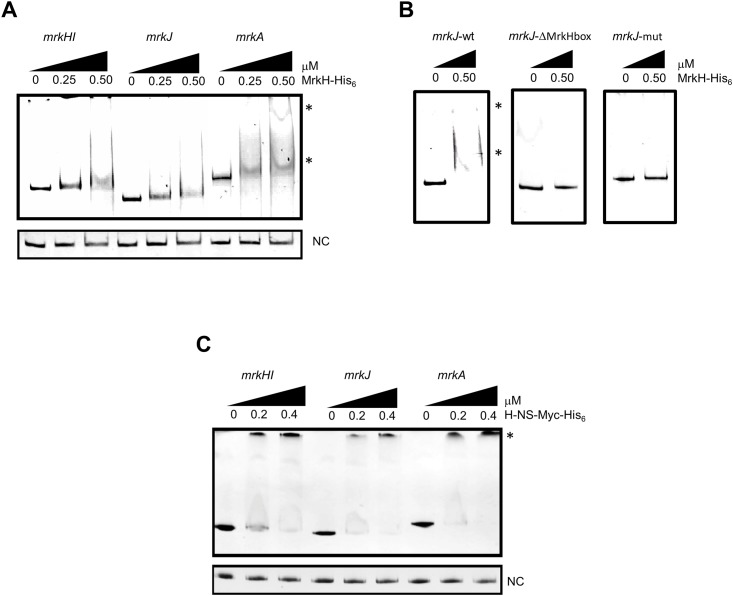
MrkH and H-NS directly bind to *mrk* genes. EMSA experiments were performed to test the binding of purified recombinant MrkH-His_6_ (A and B) or H-NS-*Myc*-His_6_ (C) proteins to the corresponding amplified DNA fragment from *mrkHI*, *mrkJ* (wt, ΔMrkHbox and mut) and *mrkA* regulatory regions. One hundred nanograms of the PCR product of each regulatory region was mixed, incubated with increasing concentrations (μM) of purified H-NS-*Myc*-His_6_ and MrkH-His_6_, and subsequently separated in 6% polyacrylamide gels. DNA-protein complexes stained with ethidium bromide are indicated (*). *fbpA* coding region of *M*. *tuberculosis* was used as negative control (NC).

To determine whether MrkH and H-NS directly regulates *mrkI* and *mrkJ*, the interaction of purified MrkH-His_6_ and H-NS-*Myc*-His_6_ proteins and DNA fragments carrying the regulatory regions of the *mrk* genes was analyzed by EMSA. The interaction of MrkH-6XHis with the regulatory regions of *mrkA/mrkH* and with a DNA fragment of *M*. *tuberculosis fbpA*, were also analyzed as positive and negative controls, respectively. MrkH-His_6_ recombinant protein specifically bound to the regulatory regions of *mrkA* and *mrkH* as previously described [21, 23] (Fig 3A). Interestingly, MrkH-His_6_ protein also interacted with the *mrkJ* regulatory region, supporting that MrkH directly activates *mrkJ* expression (Fig 3A). Both, deletion of MrkH-box or the nucleotide change of the TAT conserved motif affected the binding of MrkH-His_6_ protein on the *mrkJ* promoter (Fig 3B). These results indicate that MrkH recognizes a site located at position -63.5 relative to the transcriptional start site of *mrkJ* gene.

In addition to MrkH-His_6_ protein, we analyze whether H-NS-*Myc*-His_6_ recombinant protein could bind to the upstream region of these three promoters. We found that H-NS-*Myc*-His_6_ protein specifically bound to the *mrk* promoters (Fig 3B). These observations strongly suggest that MrkH and H-NS are regulators that directly bind to *mrk* promoters and that both proteins have antagonistic functions.

### MrkH differentially regulates *mrk* promoters

**Fig 4:**
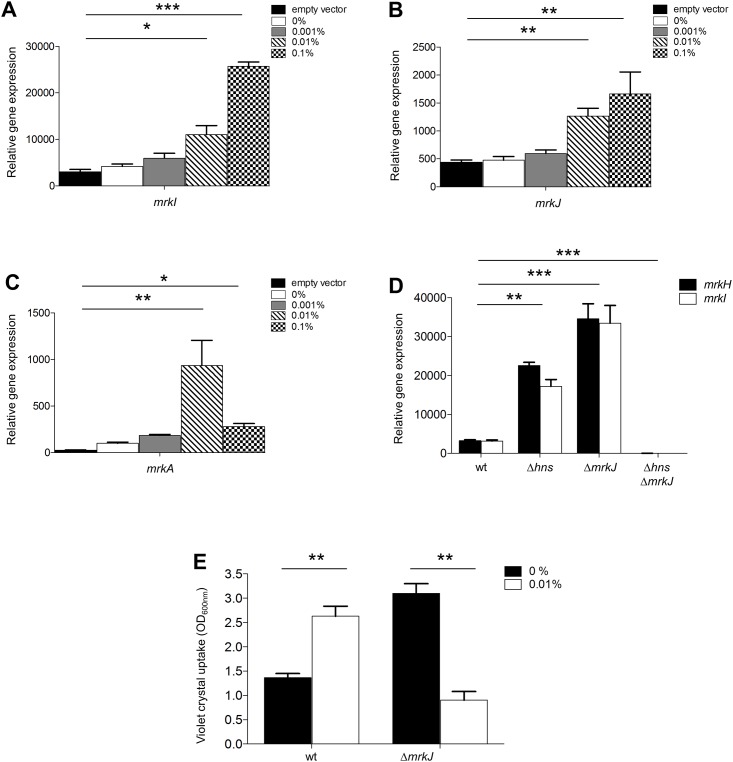
MrkH acts as repressor of *mr*k genes. Transcriptional expression (qRT-PCR) of *mrkI* (A), *mrkJ* (B) and *mrkA* (C) genes overexpressing the MrkH protein at different L(+)-arabinose concentrations in the WT strain. D. Transcriptional expression (qRT-PCR) of *mrkH* and *mrkI* in the WT, Δ*hns*, Δ*mrkJ* and Δ*hns* Δ*mrkJ* backgrounds. E. Quantification of biofilm formation by measuring violet crystal uptake under overexpression of MrkH in the WT and Δ*mrkJ* mutant. Results represent the mean and standard deviations of three independent experiments performed. Statistically significant with respect to the WT strain: *p<0.05; **p<0.01; ***p<0.001.

It has been reported that at high concentrations, antagonists of H-NS can act as transcriptional repressors [36, 38]. In order to investigate whether an over-production of MrkH protein could repress *mrk* genes, we introduced the pT6-MrkH plasmid, which expresses MrkH under an arabinose-inducible promoter, into the WT *K*. *pneumoniae* strain, to induce the expression of different amounts of MrkH with distinct concentrations of arabinose. MrkH increased the expression of the *mrkI* and *mrkJ* genes at all arabinose concentrations tested, although this was not observed for *mrkA* (Fig 4A–4C). Interestingly, *mrkA* expression reached a peak of induction at an arabinose concentration of 0.01%, while diminished at 0.1% (Fig 4C). Therefore, our results indicate that high expression levels of MrkH can repress T3P. Since Johnson and Clegg (2010) and Ares et al (2016) have shown that MrkJ and H-NS are the main negative regulators of *mrkH*, we evaluated the expression of *mrkH* and *mrkI* in several *K*. *pneumoniae* backgrounds: WT, Δ*hns* mutant, Δ*mrkJ* mutant and Δ*hns* Δ*mrkJ* double mutant. Transcriptional expressions of *mrkH* and *mrkI* were derepressed in the absence of H-NS or MrkJ, supporting the negative role of these two proteins (Fig 4D). In contrast, both *mrkH* and *mrkI* genes were repressed in the Δ*hns* Δ*mrkJ* double mutant (Fig 4D). Since MrkH autoregulates its own expression [21], our observations demonstrate that in the absence of both H-NS and MrkJ, *mrkH* expression is repressed, suggesting that high levels of MrkH could repress *mrkH* gene and subsequently the MrkH-dependent *mrk* genes. To confirm the negative role of MrkH at functional level, we evaluated its overexpression on the biofilm formation. Since a Δ*hns* mutant does not form biofilm [26], MrkH protein was overexpressed in the WT and Δ*mrkJ* mutant. The induction of MrkH stimulated the biofilm formation in the WT strain, while in the absence of MrkJ, this phenomenon was diminished (Fig 4E). These observations support the repressor activity of MrkH protein on T3P expression.

### MrkI does not affect the expression of T3P

**Fig 5:**
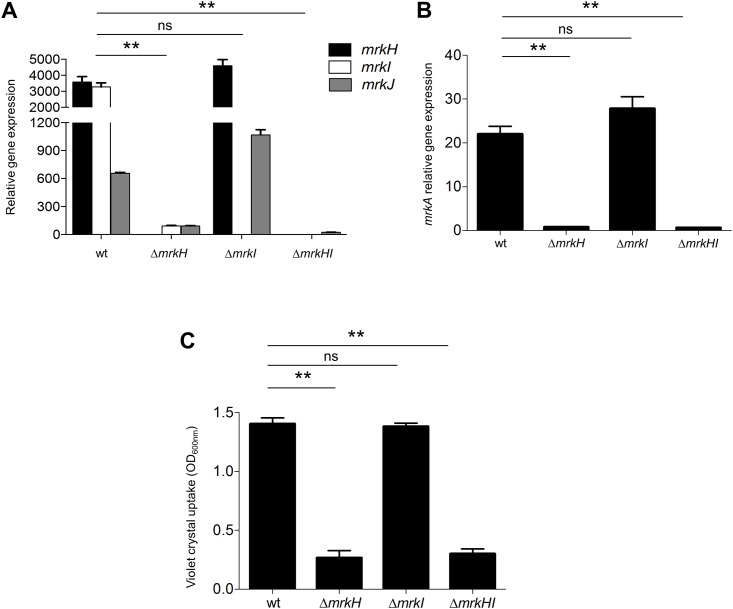
The absence of MrkI does not affect the T3P. Transcriptional expression (qRT-PCR) of *mrkH-I-J* (A) and *mrkA* (B) genes in WT, Δ*mrkH* mutant, Δ*mrkI* mutant and Δ*mrkHI* double mutant. C. Quantification of biofilm formation by measuring violet crystal uptake in WT, Δ*mrkH* mutant, Δ*mrkI* mutant and Δ*mrkHI* double mutant. Results shown represent the mean and standard deviations of three independent experiments. ns, not significant; **, statistically significant with respect to the WT strain (*p*<0.01).

*mrkI* is found adjacent to *mrkH*, and codes for a LuxR-type regulator [20]. In order to investigate whether MrkI is somehow involved in the MrkH-mediated regulation of the *mrk* genes, we determined the transcriptional expression of *mrk* genes in several backgrounds: WT, Δ*mrkH* mutant, Δ*mrkI* mutant and Δ*mrkHI* double mutant. Transcription of *mrkJ* gene was down-regulated in both Δ*mrkH* and Δ*mrkHI* mutants but not in Δ*mrkI* (Fig 5A). In addition, MrkI did not affect the expression of either *mrkH* regulatory or *mrkA* pilin genes (Fig 5A and 5B), supporting the crucial role of MrkH regulatory protein on *mrk* genes [20, 23]. In order to show that MrkI does not affect the T3P at functional level, we performed assays of biofilm formation using the same mutant strains. We found that MrkH but not MrkI affected the biofilm formation of *K*. *pneumoniae* (Fig 5C), showing the same phenotype observed in the transcriptional expression of *mrkA*. Overall, this set of data may suggest that MrkI protein is not involved in the regulation of T3P.

## Discussion

*K*. *pneumoniae* is a well-established opportunistic pathogen causing nosocomial infections. One of its most studied virulence factors is the T3P, that helps this bacterium to be adhered to both biotic and abiotic surfaces, and therefore, to establish a successful colonization in host tissues. Because of this relevant function for the pathogenesis of *K*. *pneumoniae*, this pilus must be subject to a fine regulation, not only at transcriptional level, but also at post-transcriptional and post-translational levels. In this sense, MrkH has been reported to be the master regulator of T3P. Since MrkH has a c-di-GMP-binding domain [23], its activity is controlled by fluctuations in concentrations of this second messenger. The transcriptional control of T3P is driven by three promoters located upstream from the coding regions of *mrkA*, *mrkH* and *mrkJ* genes. MrkH protein activates both *mrkABCDF* and *mrkHI* promoters, functioning as a classic transcriptional activator interacting with the α-CTD of RNA polymerase [21, 22]. Our data show that MrkH also positively regulates the *mrkJ* gene (Fig 1C), by binding to its regulatory region (Fig 3A), probably by a mechanism similar to that in the other *mrk* promoters. In terms of consensus sequence, we found that the putative MrkH-binding box on *mrkJ* promoter presented homology to that reported for *mrkH* in its auto-regulation [21]. Interestingly, a TAT motif located in the center of the MrkH-binding boxes (Fig 1B) has been reported to participate in the recognition of MrkH protein to both *mrkH* and *mrkA* promoters [21, 22]. Both MrkH-binding box deletion and site-directed mutagenesis experiments corroborated the relevance of this putative box on the MrkH-mediated *mrkJ* regulation and the *in vitro* binding of MrkH-His_6_ on DNA. Our assays of DNA-protein interaction did not include c-di-GMP; however, it has been reported that MrkH can bind *in vitro* to the promoter region of *mrk* genes in the absence of c-di-GMP [21, 23].

Whereas MrkJ indirectly represses the transcription of *mrk* genes by degrading c-di-GMP, H-NS directly silences them [25, 26], and these two proteins are considered to be the main negative regulators of T3P. Indeed, we have shown that H-NS protein binds to the three *mrk* promoters silencing their expression (Fig 3B). We showed that MrkH has a dual function: it acts as an anti-repressor of H-NS protein, antagonizing its negative effect on *mrk* genes and as a classic transcriptional activator because it is necessary to activate the expression of *mrk* genes (Fig 2). Similar to MrkH, *Vibrio cholerae* VpsT protein has also been reported to be an anti-repressor of H-NS that binds c-di-GMP, overcoming H-NS-mediated repression of biofilm genes [39, 40]. Accordingly, MrkH and VpsT are required for biofilm formation in both pathogens and they have anti-repressor/activator dual activity. Our data suggest a mechanism of competition between MrkH and H-NS on the *mrkJ* promoter, similar to what has been reported for other antagonists such as SlyA, RovA, Ler, and LeuO [36, 41–44]. We are currently trying to define the mechanistic details of how MrkH protein counteracts the H-NS-mediated repression of *mrk* promoters.

Some anti-repressors of H-NS have a negative role on several genes, and it is thought that this function may be relevant to maintain optimal levels of the proteins coded by such genes, probably to control their toxic effects. In our work, we detected high levels of *mrkH* transcription in Δ*hns* and Δ*mrkJ* single mutants; in contrast, *mrkH* expression was abolished in the Δ*hns* Δ*mrkJ* double mutant (Fig 4D). These results may suggest that the high transcriptional expression together to high concentrations of c-di-GMP may provoke that MrkH negatively auto-regulates its own expression. A similar repression pattern was detected for *mrkI* in the Δ*hns* Δ*mrkJ* double mutant (Fig 4D). Moreover, the overexpression of MrkH diminished the biofilm formation in an *mrkJ* mutant (Fig 4E), since the biofilm formation is T3P-dependent. Our experiments showed that in addition to its role as an anti-repressor and transcriptional activator, MrkH acts also as a repressor. Antagonists of H-NS such as Ler in enteropathogenic *E*. *coli* and LeuO in *Salmonella enterica*, function as concentration-dependent transcriptional repressors. In the case of Ler, a negative effect of this protein on its own auto-regulation in *LEE1* promoter has been reported [38]. LeuO, a LysR-type regulator, activates *ompS2* gene at low concentrations and represses it at high concentrations [36], where the negative effect is suggested to occur by the competition of LeuO with the OmpR transcriptional activator for the binding site. In fact, previous reports have demonstrated by EMSA experiments that different DNA-MrkH complexes are formed, suggesting that MrkH can oligomerize on the promoter region of *mrkA* [23]. According to our results, we hypothesized that high level of MrkH may cause the binding of this protein close to the -35 and -10 boxes, blocking the interaction of RNA polymerase with the promoter. Footprinting experiments will elucidate the nucleotides recognized by MrkH on *mrk* promoters.

In previous experiments our group has shown that the absence of H-NS up-regulated the expression of *mrkH*, *mrkI* and *mrkJ*, while the expression of *mrkA* is down-regulated [26]. Thus, MrkH induced in the WT strain had positive and negative effects on *mrkA* expression as compared to *mrkI* and *mrkJ* genes (Fig 4A and 4C). This repression may be due to a greater affinity of MrkH on *mrkA* regulatory region as compared to *mrkHI* and *mrkJ* promoters observed by EMSA (Fig 3A) [21–23]. However, Surface Plasmon Resonance (SPR) analysis or fluorescence anisotropy would be necessary to determine the dissociation constant (*Kd*) between MrkH protein and *mrk* promoters.

In addition to MrkH, MrkI protein is also coded in the *mrk* cluster adjacent to *mrkH*, forming a bicistronic operon [20]. Controversial results regarding the involvement of MrkI on both *mrk* expression and biofilm formation in *K*. *pneumoniae* have been reported [20, 23, 24]. While Johnson et al (2011) have reported that the absence of MrkI has significantly reduced levels of *mrkA* transcription, Wilksch et al (2011) have reported that the Δ*mrkI* mutant appears to express more *mrkA* than the WT strain. The conditions evaluated in these studies as well as the differences between *K*. *pneumoniae* strains, could possibly explain the discrepancies in the phenotypes. However, our results show that MrkI does not participate neither in the T3P expression nor in biofilm formation of *K*. *pneumoniae* (Fig 5).

In conclusion our work provides new insights into the complex regulatory functions of MrkH protein on the transcriptional control of T3P in *K*. *pneumoniae*. This wide range of MrkH function would explain the importance of intracellular concentrations of this protein to regulate *K*. *pneumoniae* virulence functions such as biofilm formation, adherence to eukaryotic cells and colonization of its host.
